# Leveraging machine learning to enhance postoperative risk assessment in coronary artery bypass grafting patients with unprotected left main disease: a retrospective cohort study

**DOI:** 10.1097/JS9.0000000000002032

**Published:** 2024-08-08

**Authors:** Ahmed Elmahrouk, Amin Daoulah, Prashanth Panduranga, Rajesh Rajan, Ahmed Jamjoom, Omar Kanbr, Badr Alzahrani, Mohammed A. Qutub, Nooraldaem Yousif, Tarique Shahzad Chachar, Youssef Elmahrouk, Ali Alshehri, Taher Hassan, Wael Tawfik, Kamel Hazaa Haider, Abdulwali Abohasan, Adel N. Alqublan, Abdulrahman M. Alqahtani, Mohamed Ajaz Ghani, Faisal Omar M. Al Nasser, Wael Almahmeed, Ahmed A. Ghonim, Shahrukh Hashmani, Mohammed Alshehri, Abdelmaksoud Elganady, Abeer M. Shawky, Adnan Fathey Hussien, Seraj Abualnaja, Taha H. Noor, Ibrahim A. M. Abdulhabeeb, Levent Ozdemir, Wael Refaat, Hameedullah M. Kazim, Ehab Selim, Issam Altnji, Ahmed M. Ibrahim, Abdullah Alquaid, Amr A. Arafat

**Affiliations:** a Department of Cardiovascular Medicine, King Faisal Specialist Hospital and Research Center, Jeddah, Kingdom of Saudi Arabia; b Department of Cardiothoracic Surgery, Faculty of Medicine, Tanta University, Egypt; c Department of Cardiology, National Heart Center, Royal Hospital, Muscat, Sultanate of Oman, Oman; d Department of Cardiology, Sabah Al Ahmad Cardiac Center, Al Amiri Hospital, Sharq, Kuwait; e Faculty of Medicine, Elrazi University, Khartoum, Sudan; f Department of Cardiology, Prince Sultan Cardiac Center, Riyadh; g Department of Medicine, Cardiology Center of Excellence, King Abdulaziz University, Jeddah, Kingdom of Saudi Arabia; h Department of Cardiology, Mohammed Bin Khalifa Specialist Cardiac Center, Awali, Kingdom of Bahrain; i Faculty of Medicine, Tanta University, Tanta, Egypt; j Department of Cardiology, College of Medicine, King Khalid University, Abha, Kingdom of Saudi Arabia; k Department of Cardiology, Bugshan General Hospital, Jeddah; l Department of Cardiology, Prince Sultan Cardiac Center, Qassim; m Department of Cardiology, King Salman Heart Center, King Fahad Medical City, Riyadh; n Department of Cardiology, Madinah Cardiac Center, Madinah, Kingdom of Saudi Arabia; o Heart and Vascular Institute, Cleveland Clinic Abu Dhabi, UAE; p Department of Cardiology, Prince Khaled Bin Sultan Cardiac Center, Khamis Mushait; q Department of Cardiology, Dr Erfan and Bagedo General Hospital, Jeddah, Kingdom of Saudi Arabia; r Department of Cardiology, Faculty of Medicine, Alazhr University, Cairo, Egypt; s Department of Cardiology, International Medical Center, Jeddah; t Department of Interventional Cardiology, King’s College London Hospital, Jeddah, Saudi Arabia; u Department of Cardiology, King Abdulaziz Specialist Hospital, Al Jawf; v Department of Cardiology, King Fahad Specialist Hospital, Tabuk; w Department of Cardiology, Prince Sultan Cardiac Center, Al Hassa; x Department of Cardiology, Alhada Armed Forces Hospital, Taif, Kingdom of Saudi Arabia; y Department of Cardiology, St James’s Hospital, Dublin, Ireland; z Department of Cardiology, Saudi German Hospital, Jeddah; aa Department of Emergency Medicine, King Abdulaziz Medical City; bb Scientific Research Center, Ministry of Defense Health Services, Riyadh, Saudi Arabia

**Keywords:** coronary artery bypass grafting, explainable artificial intelligence, left main coronary artery, machine learning

## Abstract

**Background:**

Risk stratification for patients undergoing coronary artery bypass surgery (CABG) for left main coronary artery (LMCA) disease is essential for informed decision-making. This study explored the potential of machine learning (ML) methods to identify key risk factors associated with mortality in this patient group.

**Methods:**

This retrospective cohort study was conducted on 866 patients from the Gulf Left Main Registry who presented between 2015 and 2019. The study outcome was hospital all-cause mortality. Various machine learning models [logistic regression, random forest (RF), k-nearest neighbor, support vector machine, naïve Bayes, multilayer perception, boosting] were used to predict mortality, and their performance was measured using accuracy, precision, recall, F1 score, and area under the receiver operator characteristic curve (AUC).

**Results:**

Nonsurvivors had significantly greater EuroSCORE II values (1.84 (10.08–3.67) vs. 4.75 (2.54–9.53) %, *P*<0.001 for survivors and nonsurvivors, respectively). The EuroSCORE II score significantly predicted hospital mortality (OR: 1.13 (95% CI: 1.09–1.18), *P*<0.001), with an AUC of 0.736. RF achieved the best ML performance (accuracy=98, precision=100, recall=97, and F1 score=98). Explainable artificial intelligence using SHAP demonstrated the most important features as follows: preoperative lactate level, emergency surgery, chronic kidney disease (CKD), NSTEMI, nonsmoking status, and sex. QLattice identified lactate and CKD as the most important factors for predicting hospital mortality this patient group.

**Conclusion:**

This study demonstrates the potential of ML, particularly the Random Forest, to accurately predict hospital mortality in patients undergoing CABG for LMCA disease and its superiority over traditional methods. The key risk factors identified, including preoperative lactate levels, emergency surgery, chronic kidney disease, NSTEMI, nonsmoking status, and sex, provide valuable insights for risk stratification and informed decision-making in this high-risk patient population. Additionally, incorporating newly identified risk factors into future risk-scoring systems can further improve mortality prediction accuracy.

## Introduction

Highlights

- Traditional cardiac surgery scoring system (EuroSCORE II) performed well for predicting mortality after coronary artery bypass grafting for unprotected left main coronary disease; however, machine learning (ML) methods offered even better accuracy.
- Random forest achieved the best results in predicting patient mortality in this group.
- ML analysis identified the most important factors to predict mortality, including preoperative lactate level, emergency surgery, and chronic kidney disease.
- Thorough model performance evaluation with several performance metrics is required for studies reporting results of ML analysis.

Risk-scoring systems in cardiac surgery are essential tools for assessing the likelihood of surgical outcomes^1^. Additionally, risk scores guide patients’ counseling, treatment choices, and quality improvement^1–3^. Coronary artery bypass grafting (CABG) remains the primary treatment option for left main coronary artery (LMCA) disease; however, accurate assessment of surgical risk is essential for informed decision-making^4^. While risk-scoring systems such as the European System for Cardiac Operative Risk Evaluation (EuroSCORE) and the Society of Thoracic Surgeons (STS) score have become mainstays in cardiac surgery, their limitations are increasingly recognized. These limitations stem from their reliance on historical data, potentially neglecting advancements in surgical techniques and technology and including patients with specific ethnicities. Moreover, traditional risk stratification methods are limited by the use of ordinary data analysis techniques, which are affected by high-dimensional and sparse data. As a result, the accuracy and generalizability of these scores for predicting outcomes in specific patient populations, such as those from the Gulf region, can be compromised^1,5^.

The future of risk-scoring in cardiac surgery lies in incorporating novel variables such as genetic data, advanced laboratory results, and sophisticated imaging data. Machine learning (ML) offers a powerful approach to unlock this potential. ML algorithms excel at uncovering complex relationships between variables, potentially revealing insights that traditional methods might miss^6^. They can also effectively handle large, intricate datasets and pinpoint the variables with the strongest influence on outcomes. Unlike static scoring systems, ML models continuously learn and improve by integrating new data^7^. This dynamic approach holds immense promise for achieving more accurate and personalized risk stratification for cardiac surgery patients.

While prior research has demonstrated the promise of ML in predicting outcomes after CABG, with high accuracy compared to traditional scoring systems^8–10^, a gap remains in its application for specific patient populations. In the age of big data, there is a growing need for tailored risk scores that consider the intricacies of individual cardiac procedures and pathologies. This study specifically addressed a significant need by investigating the use of ML to predict hospital mortality after CABG for patients with unprotected LMCA disease, a scenario with limited existing data. The primary aim of this study was to evaluate the performance of ML models for predicting hospital mortality risk in patients with unprotected LMCA undergoing CABG. By leveraging ML algorithms, we sought to identify the key clinical, demographic, and laboratory features that are most predictive of hospital mortality in this patient group using a range of supervised ML algorithms.

## Patients and methods

### Design and patients

Included patients. This study is a part of a retrospective cohort study was conducted in three countries and included consecutive patients who underwent CABG for unprotected LMCA disease (presented between 2015 and 2019^11^. During this study period, 2657 patients with left-main coronary artery disease were presented. The study included adult patients (>18 years) who underwent CABG as the revascularization strategy for managing the unprotected LMCA disease. We excluded patients with protected LMCA disease (*n*=174), missing data (*n*=50), previous LMCA intervention (*n*=37), patients who were treated medically (*n*=193) or had percutaneous coronary artery intervention (PCI) (*n*=1222) and those who had concomitant surgery (*n*=115). After the exclusion of those patients, 866 patients were included in the final analysis.

### Study data and endpoint

Preoperative data included sex (male, female), age (in years), BMI (kg/m^2^), smoking status, diabetes mellitus status, hypertension status, dyslipidemia status, history of coronary artery disease (CAD), previous myocardial infarction (MI), history of percutaneous intervention (PCI), history of CABG, chronic kidney disease (CKD), peripheral arterial disease (PVD), cerebrovascular accident (CVA), atrial fibrillation (AF), chronic heart failure (CHF), presentation with arrest, shock, arrhythmia, ST-elevation myocardial infarction (STEMI), non-ST-elevation myocardial infarction (NSTEMI), ejection fraction (EF %), pulmonary artery systolic pressure (PASP, mmHg), valve lesions, distal LMCA disease, number of vessels with significant lesions, laboratory data on presentation (troponin, hemoglobin, and creatinine clearance), preoperative lactate, and surgical urgency. Preoperative risk stratification was performed using EuroSCORE II^12^. CKD was defined based on creatinine clearance (<90–60 ml/min)^13^. The study’s primary endpoint was hospital mortality, which was defined as mortality occurring within the first 30 days of surgery or during hospitalization Details about the G-LM registry were previously published^14–16^.

### Ethical considerations and registration

Data collection for the study was approved by the local Institutional Review Board (IRB) on 12 November 2020 and was also approved by the ethical committees of each participating centers. This was carried out in accordance with the local guidelines and ethical guidelines of the Declaration of Helsinki 2013^17^. The need for patient consent was waived because of the retrospective design. The study is registered in the Research Registry and was reported according to the strengthening the reporting of cohort, cross-sectional, and case–control studies in surgery (STROCSS) (Supplemental Digital Content 1, http://links.lww.com/JS9/D280) guidelines^18^.

### Data analysis

#### Statistical analysis

This study analyzed data to assess factors affecting hospital mortality after CABG for LMCA disease. Continuous data (normally distributed) are summarized by means and SD, while non-normally distributed data are described by medians and interquartile ranges (IQRs). Normality was assessed using the Shapiro–Wilk test. Student’s *t*-test or the Wilcoxon rank-sum test was used to compare continuous variables between survivors and nonsurvivors. Categorical data are presented as frequencies and percentages and were compared using the *χ* ^2^ test or Fisher’s exact test. Logistic regression with receiver operating characteristic (ROC) curve analysis was used to assess the predictive ability of the EuroSCORE II. Patients with missing data were omitted from the analysis.

#### Machine learning

**Figure 1:**
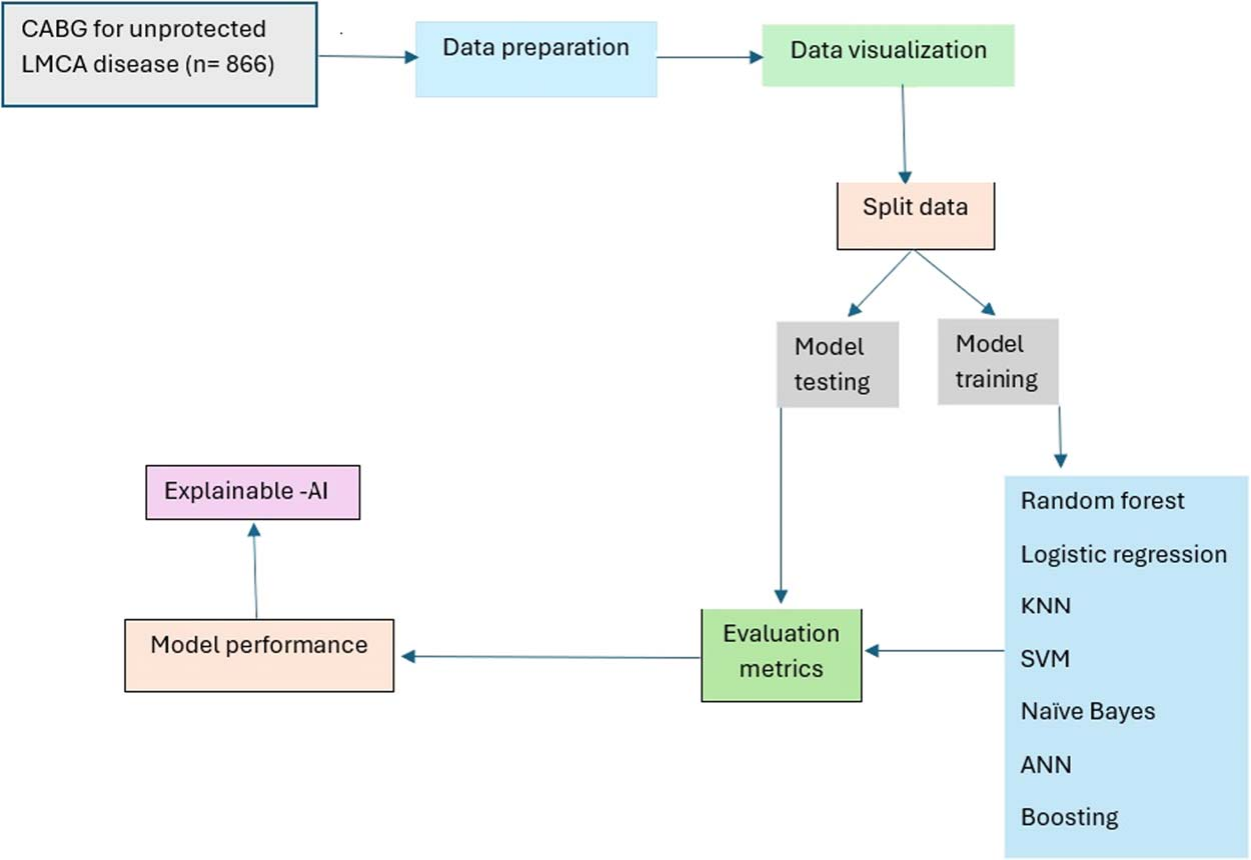
The study flow diagram showing the steps for building machine learning models. AI, artificial intelligence; ANN, artificial neural network; CABG, coronary artery bypass grafting; KNN, k-nearest neighbor; LMCA, left main coronary artery; SVM, support vector machine.

For machine learning, the data were split into training and testing sets (70%/30%). Correlations between variables were evaluated using a heatmap, and highly correlated features were excluded. Various machine learning models (logistic regression, random forest, k-nearest neighbor, support vector machine, naïve Bayes, multilayer perception, AdaBoost, CatBoost, light gradient boost, and XGBoost) were used to predict mortality, and their performance was measured by accuracy, precision, recall, F1 score, and AUC. The best-performing model’s output was interpreted using explainable artificial intelligence, namely, Shapley Additive Explanations (SHAP) and Quantum Lattice (QLattice)^19^. Only patients with complete data were included in the analysis. The dataset did not have any missing values. Study flow diagram showing the steps for building machine learning models is shown in Figure 1. Stata 18 and Python were the primary software tools used for data analysis.

## Results

### Comparison between survivors and nonsurvivors

Hospital mortality occurred in 56 patients (6.5%). Patients who died in the hospital were older and were more likely to be females and have CKD, AF, and CHF. Patients who presented with cardiac arrest, shock, or arrhythmia were significantly more likely to be nonsurvivors, and they had significantly greater preoperative lactate levels and lower creatinine clearance. Nonsurvivors more frequently underwent emergency surgery (Table 1).

**Table 1:** Comparison between hospital survivors and nonsurvivors after coronary artery bypass grafting for patients with unprotected left main disease.

	Survived (*n*=810)	Nonsurvived (*n*=56)	*P*
Female	107 (13.21%)	15 (26.79%)	0.005
Age (years)	61.11±9.72	65.27±10.77	0.002
BMI (kg/m^2^)	27.69 (24.97–31.18)	27.04 (24.22–28.80)	0.056
Smoking	335 (41.36%)	16 (28.57%)	0.059
Diabetes mellitus	586 (72.35%)	44 (78.57%)	0.312
Dyslipidemia	557 (68.77%)	43 (76.79%)	0.208
Hypertension	558 (68.89%)	45 (80.38%)	0.071
History of CAD	286 (35.31%)	25 (44.64%)	0.159
Myocardial infarction	172 (21.23%)	15 (26.79%)	0.329
Previous PCI	147 (18.15%)	13 (23.21%)	0.345
Previous CABG	10 (1.23%)	0	>0.99
Chronic kidney disease	96 (11.85%)	20 (35.71%)	<0.001
Peripheral arterial disease	55 (6.79%)	6 (10.71%)	0.267
Cerebrovascular accident	40 (4.94%)	2 (3.57%)	>0.99
Atrial fibrillation	20 (2.47%)	7 (12.50%)	<0.001
Chronic heart failure	53 (6.54%)	10 (17.86%)	0.002
Arrest	6 (0.74%)	4 (7.14%)	0.002
Shock	16 (1.98%)	9 (16.07%)	<0.001
Arrhythmia	29 (3.58%)	8 (14.29%)	<0.001
STEMI	153 (18.89%)	13 (23.21%)	0.426
NSTEMI	403 (49.75%)	31 (55.36%)	0.417
Ejection fraction (%)	50 (40–55)	50 (45–55)	0.054
PASP (mmHg)	25 (20–32)	30 (20–42)	0.104
Aortic stenosis	12 (1.48%)	3 (5.36%)	0.067
Aortic regurgitation	9 (1.11%)	0	>0.99
Mitral stenosis	2 (0.25%)	1 (1.79%)	0.182
Mitral regurgitation	95 (11.73%)	11 (19.64%)	0.081
Tricuspid regurgitation	30 (3.70%)	5 (8.93%)	0.069
Distal left main disease	611 (75.43%)	45 (80.36%)	0.406
Number of vessels	3 (2–3)	3 (3–4)	0.337
Troponin (ng/ml)	6.38 (0.34–224)	8.5 (1.25–1564)	0.140
Lactate (mmol/l)	2.4 (1.3–4.3)	8.33 (4.45–11)	<0.001
Hemoglobin (mg/dl)	13.2 (11.7–14.4)	12.9 (10.6–14.5)	0.352
Creatinine clearance (ml/min)	83 (70–90)	70 (36–90)	0.001
Emergency surgery	161 (19.88%)	25 (44.64%)	<0.001

The data are presented as the means, SD, medians, IQRs, or numbers and percentages.

CABG, coronary artery bypass grafting; CAD, coronary artery disease; NSTEMI, non-ST-segment elevation myocardial infarction; PASP, pulmonary artery systolic pressure; PCI, percutaneous coronary intervention; STEMI, ST-elevation myocardial infarction.

### EuroSCORE prediction of mortality

**Figure 2:**
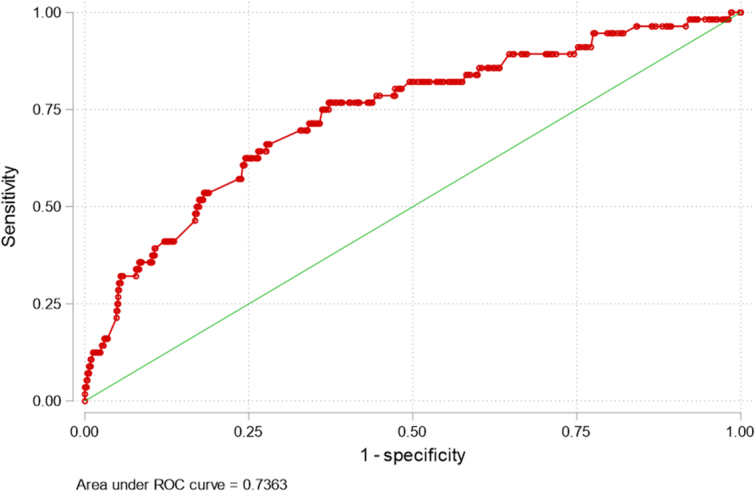
Receiver operating characteristic (ROC) curve of the association between EuroSCORE II and hospital mortality. The area under the curve (AUC) is 0.736.

Nonsurvivors had significantly greater EuroSCORE II values (1.84 (10.08–3.67) vs. 4.75 (2.54–9.53) %, *P*<0.001 for survivors and nonsurvivors, respectively). The EuroSCORE II score significantly predicted hospital mortality (OR: 1.13 (95% CI: 1.09–1.18), *P*<0.001), with an AUC of 0.736 (Fig. 2).

### Machine learning algorithms

The heatmap showed a strong correlation between STEMI and emergency surgery; therefore, STEMI was omitted from the analysis. Furthermore, a correlation was found between a history of CAD, previous PCI, and previous MI. Only previous MIs were included in the machine learning analysis (Supplementary Figure 1, Supplemental Digital Content 2, http://links.lww.com/JS9/D281).

**Figure 3:**
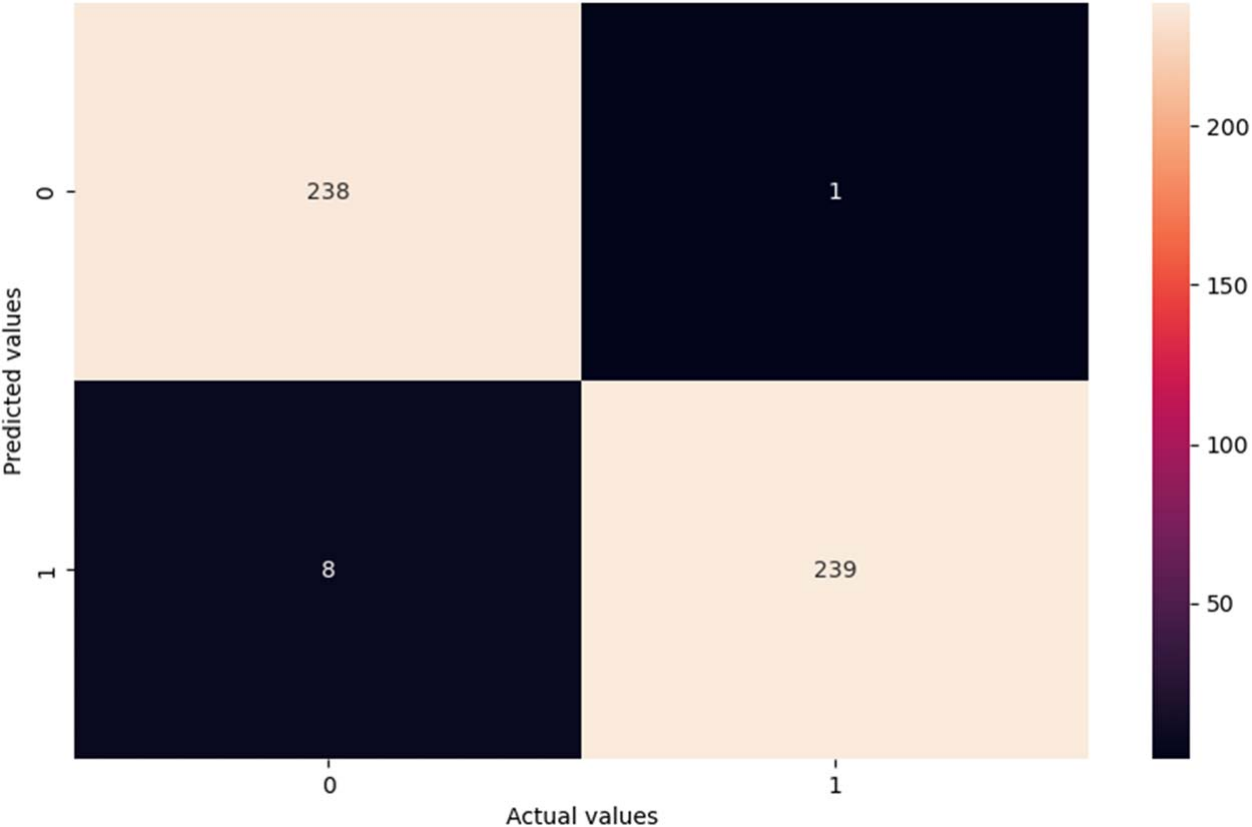
Confusion matrix of the random forest model predicting in-hospital mortality after CABG for unprotected left main coronary artery disease.

The performances of the different machine learning models are presented in Table 2. The random forest algorithm achieved the best performance parameters (Fig. 3) (Supplementary Figure 2, Supplemental Digital Content 3, http://links.lww.com/JS9/D282). The maximum depth of the random forest was 90, the maximum number of features was 3, the minimum sample leaf was 3, the minimum sample split was 8, and the number of estimators was 300.

**Table 2:** The accuracy, precision, recall, F1 score, and area under the curve (AUC) for the performance of machine learning algorithms used to predict hospital mortality.

Model	Accuracy (%)	Precision (%)	Recall (%)	F1 (%)	AUC (%)
Random Forest	98	100	97	98	100
Logistic regression	93	0	0	0	79
KNN	93	0	0	0	62
SVM	92	92	92	92	96
Naïve Bayes	55	53	100	69	91
Multilayer perception (ANN)	93	0	0	0	50
AdaBoost	94	56	29	38	78
CatBoost	93	50	6	11	81
Light gradient boost	93	50	6	11	80
XgBoost	94	100	6	11	84

ANN, artificial neural network; KNN, k-nearest neighbor; SVM, support vector machine.

### Explainable artificial intelligence

**Figure 4:**
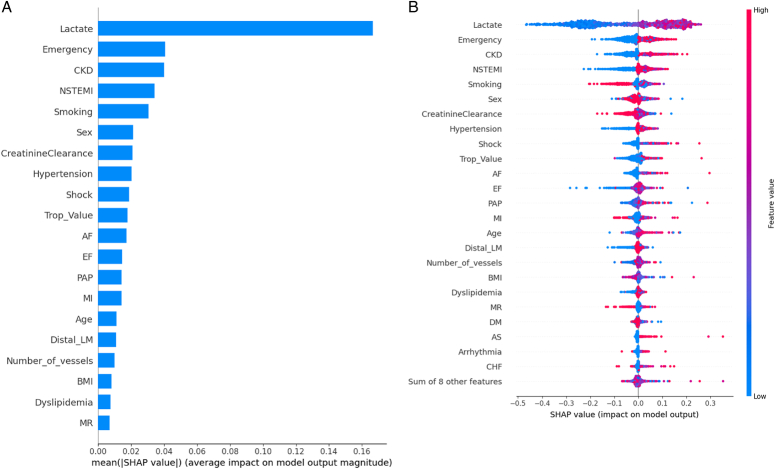
(A) Feature importance ranking in the random forest model for predicting in-hospital mortality after CABG for left main coronary artery disease using SHAP (B) SHAP feature importance for predicting in-hospital mortality after CABG. Red indicates a greater SHAP value (greater influence on prediction), and the position on the *X*-axis represents the feature value (higher on the right). Positive SHAP values increase mortality prediction, while negative values increase survival prediction. AF, atrial fibrillation; CKD, chronic kidney disease; EF, ejection fraction; LM, left main; MI, myocardial infarction; MR, mitral regurgitation; NSTEMI, non-ST-elevation myocardial infarction; PAP, pulmonary artery pressure; Trop, troponin.

**Figure 5:**
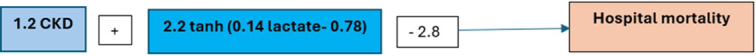
Quantum graph for factors predicting hospital mortality in CABG patients with left main coronary artery disease. CKD, chronic kidney disease.

Explainable artificial intelligence was used to interpret the reasoning behind the model performance. A bar plot was used to demonstrate the importance of each feature. The bar plot arranged factors affecting hospital mortality according to importance were as follows: preoperative lactate level, emergency surgery, chronic kidney disease, NSTEMI, smoking status, sex, creatinine clearance status, hypertension status, shock status, troponin levels, AF, ejection fraction, pulmonary artery systolic pressure, previous MI, age, distal left main disease status, number of vessels with significant disease, BMI, dyslipidemia status, and presence of mitral regurgitation (Fig. 4A). In the beeswarm plot, red color indicates a high value, and positive values indicate increasing mortality. The most important features identified by the beeswarm plot were lactate, emergency surgery, CKD, NSTEMI, smoking status, and female sex. Smoking increased survival (Fig. 4B). QLattice identified lactate and CKD as the two most important factors for predicting hospital mortality in CABG for unprotected LM disease patients. The Q graph can be interpreted as follows: logreg (mortality)=(1.2 CKD+ 2.2 tanh (0.14 lactate-0.78)-2.8) (Fig. 5).

## Discussion

Machine learning is rapidly transforming healthcare by enabling the prediction of disease outcomes using vast amounts of data. These predictions can significantly impact clinical decision-making, paving the way for personalized medicine and ultimately improving overall healthcare quality^20^. Compared to traditional statistical methods, machine learning offers several advantages. Machine learning algorithms excel at handling complex and voluminous data, uncovering nuanced patterns and relationships that might otherwise be missed. Additionally, they have the ability to continuously learn and improve as they are exposed to more data. This inherent adaptability allows them to keep pace with evolving medical practices and changes in patient populations over time^21^.

Risk-scoring systems have become integrated into daily medical practice to guide treatment and patient counseling. Machine learning methods can enhance the performance of risk-scoring systems by improving variable selection, predictive power, and precision^22^. Previous studies have demonstrated the superior performance of machine learning models in predicting outcomes after CABG^8,23^. It became necessary to evaluate the risk factors for each cardiac procedure and disease separately, especially in this era of big data. However, data predicting hospital mortality after CABG for unprotected LMCA disease are still lacking. This study evaluated the ability of the EuroSCORE to predict mortality and tested the performance of several machine learning models in those patients. The EuroSCORE II score significantly predicted mortality, with an AUC of 0.736. Random forest had the best model performance parameters, with an accuracy of 98%.

Our study highlights the potential impact of sample size on machine learning performance, particularly for rare events such as hospital mortality after CABG for unprotected LMCAs. This finding is supported by the findings of Khalaji *et al*.^9^, who included 16 850 patients and 468 patients who died. The logistic regression performed best (AUC: 0.81), followed by XGBoost (AUC: 0.79), naïve Bayes (AUC: 0.78), random forest (AUC: 0.78) and support vector machine (AUC: 0.74). The most important features that affected the outcomes were the number of ventilation hours and left ventricular ejection fraction. In a study on the prediction of bleeding after CABG using machine learning, random forest had the highest AUC (0.83), followed by stochastic gradient boosting (AUC: 0.81)^24^. Zhang and associates evaluated machine learning in predicting severe complications after off-pump CABG^25^. The XGBoost model achieved the highest AUC (0.94) and highest accuracy; however, the SVM had the lowest AUC (0.75). CatBoos had an AUC of 81 in this study, while the precision was 50%. This finding indicates the importance of thorough model evaluation and the use of a confusion matrix, particularly if the event number is low.

Explainable AI techniques revealed that preoperative lactate level, emergency surgery, CKD, NSTEMI, and female sex were the most important features for predicting hospital mortality. Interestingly, smoking appeared to be associated with improved survival. The quantum lattice analysis further highlighted the significance of preoperative lactate levels and CKD in mortality prediction. Notably, neither lactate levels nor patient presentation are currently incorporated into EuroSCORE II. Although the impact of intraoperative and postoperative lactate levels on cardiac surgery mortality has been established^26–28^, data on preoperative lactate levels are limited. This finding is particularly relevant for LMCA disease patients, who often present with cardiac arrest or shock and undergo emergency intervention.

The effect of smoking on CABG outcomes remains controversial^29–32^. Our study revealed a ‘smoker’s paradox’, where smoking was associated with improved survival. This finding aligns with the findings of Tang *et al*.^33^, who reported that current smokers with left ventricular dysfunction had lower mortality after CABG than nonsmokers did. The random forest model also identified factors not included in EuroSCORE II, such as hypertension, shock, troponin level, and atrial fibrillation. Integrating these variables into future risk stratification models for LMCA disease patients has the potential to enhance their accuracy.

### Future perspectives

The findings of this study suggest that EuroSCORE II, while useful, may benefit from the incorporation of ML methods to enhance mortality prediction after CABG for LMCA disease. ML algorithms, particularly random forests, demonstrated improved accuracy compared to traditional scoring systems. However, the wide variability in ML model performance highlights the challenge of working with small event numbers. Research on machine learning methods with small event numbers should explore alternative performance indicators other than the AUC. Moreover, the identification of novel risk factors emphasizes the need to update existing scoring systems to improve their predictive capabilities. This study demonstrated the potential use of ML in personalized medicine for patients undergoing CABG for LMCA disease that confirmed other studies findings^34^.

### Study limitations

While the study provides valuable insights into mortality prediction after CABG for LMCA disease, there are several limitations that should be acknowledged. The study utilized a retrospective design, which may introduce inherent limitations such as selection bias. Although the study is a multicenter study, it was conducted on a population of the same ethnicity, and the results may not be generalizable to other patients. Therefore, external validation is required to test whether the prediction is accurate for patients of different ethnicities. The small number of events also limited the study; however, random forest performance was high. Despite dividing the sample into training and testing datasets, the findings of the study were not externally validated using an independent dataset of different populations and healthcare settings. Furthermore, the study focused on CABG patients with LMCA disease, and the generalizability of the findings to other patients or procedures needs further study. It is also crucial to consider other variables that could affect hospital mortality and were not included in the analysis. Unmeasured or unknown confounders may influence the observed associations.

## Conclusions

This study provides valuable insights into the future direction of mortality prediction after CABG for LMCA disease. The findings suggest that ML methods, particularly random forests, hold promise for enhancing the accuracy of mortality prediction. However, caution should be exercised when working with small event numbers, and comprehensive model evaluation should be considered. Additionally, the incorporation of newly identified risk factors into future risk-scoring systems can further improve mortality prediction accuracy. These future perspectives have the potential to optimize patient outcomes and inform clinical decision-making in the field of cardiac surgery.

## Ethical approval

King Faisal Specialized Hospital and Research Center-Jeddah-Saudi Arabia.

In addition to local approvals from participating centers.

IRB N0. 2022-81.

## Consent

The study is retrospective in design and the IRB waived the need to have patients consent.

## Source of funding

None.

## Author contribution

A.A.A., A.E., A.D., P.P., A.J., N.Y., W.A., M.A.Q., M.A., A.E., T.H.: conceptualization, formal analysis, and writing – original draft; R.R., O.K., B.A., T.S.C., Y.E., A.A., W.T., K.H.H., A.A., A.N.A., A.M.A., M.A.G., F.O.M.A.N., A.A.G., S.H., A.M.S., A.F.H., S.A., T.H.N., I.A.M.A., L.O., W.R., H.M.K., E.S., I.A., A.M.I., and A.A.: data curation, software, supervision, and visualization. All authors contributed in investigation, methodology, writing – review and editing, and have read and agreed on the content of this manuscript.

## Conflicts of interest disclosure

None.

## Research registration unique identifying number (UIN)

The study is retrospective chart review, with no active patients recruitment; therefore, it was not registered in public domains.

## Guarantor

Amin Daoulah, MD.

## Data availability statement

The data used in this analysis is available upon reasonable requests to the corresponding author after approval of the local institution to release the data.

## Supplementary Material
