# Supplementary figures and images for: Leveraging machine learning to enhance postoperative risk assessment in coronary artery bypass grafting patients with unprotected left main disease: a retrospective cohort study
Source: Int J Surg. 2024 Aug 8;110(11):7142–9. doi: 10.1097/JS9.0000000000002032 (PMC11573096; doi:10.1097/JS9.0000000000002032)

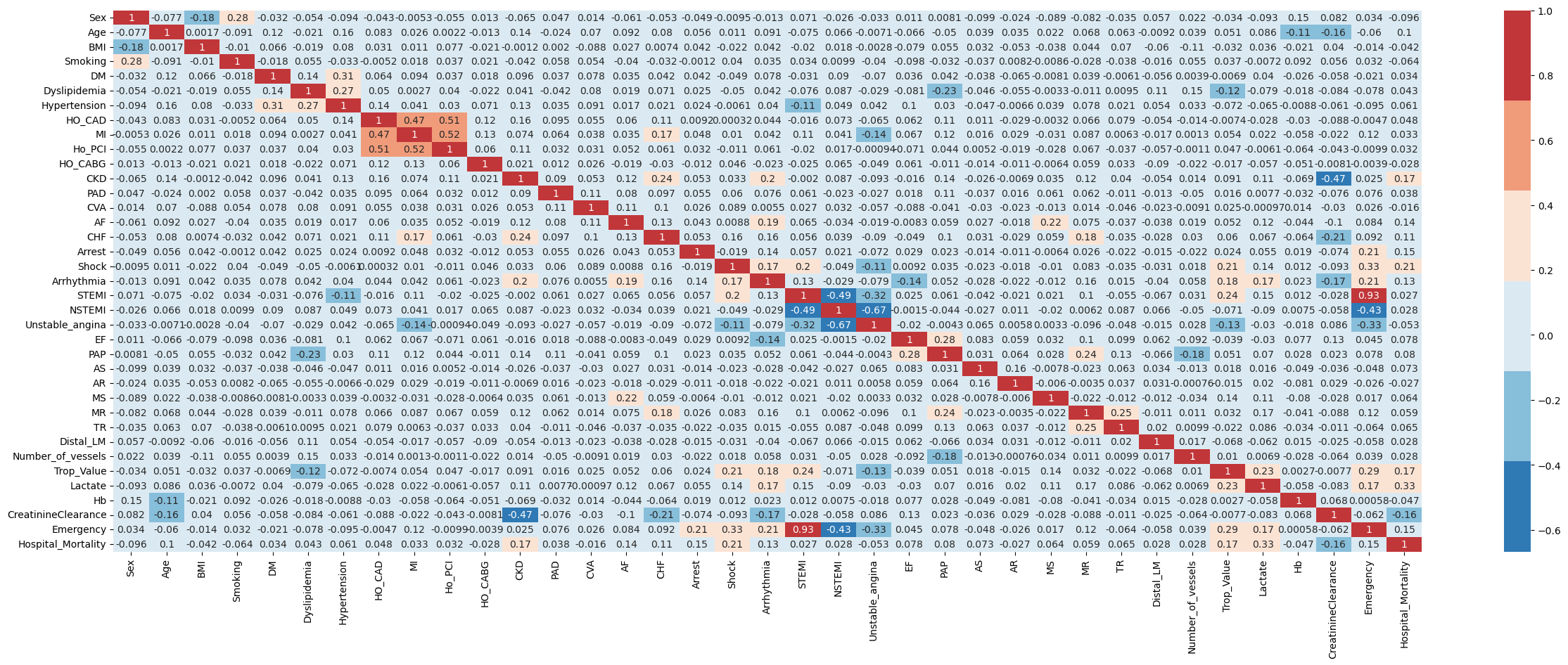


**Supplementary Figure 1:** Heatmap of correlation coefficients between variables

Supplement: Supplementary file 2 [file js9-110-7142-s002.docx]
