# Supplementary material for: Leveraging machine learning to enhance postoperative risk assessment in coronary artery bypass grafting patients with unprotected left main disease: a retrospective cohort study
Source: Int J Surg. 2024 Aug 8;110(11):7142–9. doi: 10.1097/JS9.0000000000002032 (PMC11573096; doi:10.1097/JS9.0000000000002032)
Supplement: Supplementary file 3 [file js9-110-7142-s003.docx]

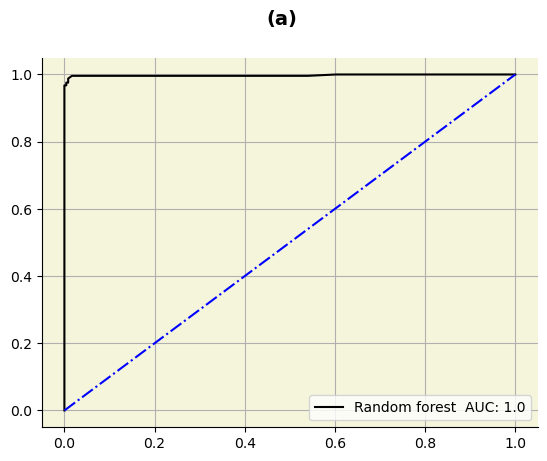


(A)


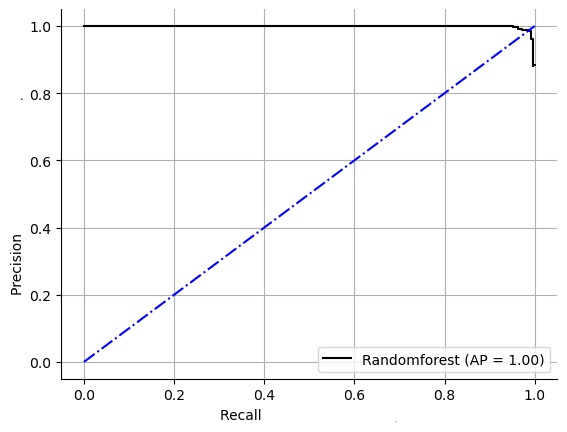


(B)

**Supplementary Figure 2:** (A) Area under the curve of the receiver operator curve of random forest model (B) precision-recall curve of random forest model
